# The Recovered Independent Ambulation Rate and Prognostic Factors of Non-ambulatory Patients After Metastatic Spinal Cord Compression Surgery

**DOI:** 10.7759/cureus.64458

**Published:** 2024-07-13

**Authors:** Keiichiro Iida, Toshiaki Sugita, Toshifumi Fujiwara, Hirokazu Saiwai

**Affiliations:** 1 Orthopaedic Surgery, Faculty of Medical Sciences, Kyushu University, Fukuoka, JPN

**Keywords:** metastatic spinal cord compression, surgery, prognosis, metastasis, ambulation

## Abstract

Purpose: Surgery could regain the ability to walk even in non-ambulatory patients with spinal cord compression due to metastatic spine disease. However, many patients cannot reach the stage of independent ambulation because most are at an advanced disease stage. This study investigated the regained independent ambulation rate after surgery and prognostic factors for independent ambulation after metastatic spinal cord compression surgery.

Methods: In a retrospective cohort study, 38 non-ambulatory patients with spinal metastases at the cervical or thoracic lesions, who underwent surgery, were included. All surgeries were performed using laminectomy and posterior fixation. Recovery rates of independent ambulation and its prognostic factors were examined. Independent ambulation was defined as the use of a walking aid without wheelchair requirement. Factors, including age, tumor type, visceral organ metastasis, past systematic cancer therapy, neurological grade, the time from leg-symptom onset to non-ambulatory stage, and the time from non-ambulatory stage to surgery, were investigated.

Results: The regained independent ambulation rate was 18% (7/38). Compared to non-ambulatory patients, those who regained independent ambulation were more likely to have less past systematic therapy (14% [1/7] vs. 74% [23/31], P=0.003) and slow paralysis progression (over seven days from leg-symptom onset to non-ambulatory stage) (86% [6/7] vs. 23% [7/31], P=0.002).

Conclusions: Recovery to independent ambulation in non-ambulatory patients with metastatic spinal cord compression was poor, even if surgery was performed. Absence of past systematic therapy and slow paralysis progression were favorable factors for regaining independent ambulation.

## Introduction

Spinal metastasis is often observed in the advanced stages of malignant tumors and sometimes causes neurological deterioration with spinal cord compression [1]. The effectiveness of surgery for the neurological deficit caused by spinal metastasis has been investigated in several reports [2,3]. Surgery could provide prolonged walking ability and even help recover the ability to walk in non-ambulatory patients with spinal cord compression. However, many non-ambulatory patients cannot be discharged as ambulatory after surgery because most are at an advanced disease stage. While walking a few steps might be possible postoperatively, recovery is often temporary and does not reach the level needed to improve performance status. In addition to neurological recovery, the general condition and disease progression influence the ambulation stage. To date, several reports have investigated the recovery rates of ambulation [2-5]. However, the definition of ambulation includes several walking conditions, such as being able to walk for a few steps [5]. Whether non-ambulatory patients could return to daily life with an independent gait after surgery remains uncertain.

This study examined the rate of recovered independent ambulation and neurological improvement after surgery to evaluate the effectiveness and limitations of surgery for non-ambulatory patients with metastatic spinal cord compression. We defined independent ambulation as a walking condition in which a walking aid could be used but a wheelchair was not required in daily life, and neurological improvement as a one-stage improvement in the modified Frankel grade [6].

## Materials and methods

We retrospectively investigated 110 consecutive cases that received surgery for spinal metastasis at our institutions between 2004 and 2020. The study included only the patients who could not walk by myelopathy, but not by radiculopathy or back pain. Sixteen patients who underwent lumbar surgery and 57 ambulatory patients were removed. The remaining 38 non-ambulatory patients with spinal metastasis at the cervical or thoracic lesions were included. No patient could walk more than a few steps, even with a walking aid for neurological deterioration. Metastatic spine and spinal cord compression were confirmed by magnetic resonance imaging (MRI). The cerebrospinal fluid was not confirmed by the compression of metastatic tumors in all cases (Bilsky grade 3) [7]. Surgery was performed with a laminectomy and posterior fixation using a spinal instrument. The tumor around the spinal cord was removed to the extent possible. Additional preoperative radiotherapy was performed as much as possible, as the patient’s condition allowed. We examined the surgical outcomes (recovery rate of independent ambulation and neurological improvement) and prognostic factors of independent ambulation. Independent ambulation was defined as the ability to walk with the use of a walking aid, but not a wheelchair, in daily life. Neurological improvement was defined as a one-stage improvement in the modified Frankel grade. Prognostic factors, including age (younger than 65 years or not), tumor type (slow, moderate, or rapid according to the modified scoring system of Katagiri et al. [8]), visceral organ metastasis (presence or absence), past history of systematic therapy for cancer (presence or absence), neurological grade (possible knee flexion or not), time from leg-symptom onset to the non-ambulatory stage (within seven days or not), and the time to surgery from the non-ambulatory stage (within 48 hours or not) were compared between patients with and without postoperative ambulation.

This study was conducted in accordance with the principles of the Declaration of Helsinki. Kyushu University Institutional Review Board issued approval 22098-00. The Chi-squared test and Wilcoxon signed-rank test were used for group comparisons. Kaplan-Meier survival curves were used to analyze postoperative survival. P-values <0.05 were considered significant for all statistical tests. All statistical analyses were performed using JMP® 16 software (SAS Institute Inc., Cary, NC, USA).

## Results

The average follow-up period was 371 days (range, 11-2028). We followed 22 of 38 patients until their deaths from the disease. The median overall survival was 482 days (range, 30-1700). The six-month and one-year survival rates were 67% and 54%, respectively. The background characteristics of the patients (24 men and 14 women) are shown in Table 1.

**Table 1:** Patients’ characteristics. SD; standard deviation

Parameter		
Age (mean±SD)		64±10
Sex (n)	male	24
	female	14
Tumor type (n)	lung	7
	prostate	5
	breast	4
	colon	4
	kidney	4
	liver	3
	sarcoma	3
	pancreas	2
	hematopoietic	2
	thyroid	1
	others	3
Location (n)	cervical	2
	thoracic	36
Past history of treatment (n)	no	14
	yes	24
Visceral organ metastasis (n)	no	18
	yes	20
Paralysis (modified Frankel grade) (n)	A	2
	B	4
	C1	18
	C2	14

The mean age was 65 years (range 41-78), and spinal metastasis sites included two cervical and 36 thoracic lesions. Primary tumors included lung (n=7), breast (n=5), prostate (n=4), colon and rectal (n=4), renal (n=4), liver (n=3), sarcoma (n=3), pancreas (n=2), hematopoietic system (n=2), thyroid (n=1), and others (n=3). Visceral organ metastasis was present in 53% (20/38) of the patients. There was a history of systematic therapy for cancer at surgery time in 63% (24/38) of the patients. The neurological status was Frankel A (n=2), Frankel B (n=3), and Frankel C (n=29) (C1:16, C2:13).

The independent ambulation and neurological recovery rates were 18% (7/38) and 58% (22/38), respectively. Patients who recovered independent ambulation were more likely to have less past systemic treatment (14% [1/7] vs. 26% [8/31], P=0.003) and a slow progression of neurological symptoms (over seven days from leg-symptom onset to the non-ambulatory stage; 86% [6/7] vs. 23% [7/31], P=0.002) (Table 2).

**Table 2:** Comparison of prognostic factors between ambulatory and non-ambulatory patients.

	Ambulatory (7)	Non-Ambulatory (31)	P-value
Age (<65 years), n (%)	1(14)	14(45)	0.13
Visceral organ metastasis (+), n (%)	3(33)	17(55)	0.33
Past history of therapy (+), n (%)	1(14)	23(74)	0.003
Neurological grade (possible knee up), n (%)	3(43)	11(35)	0.71
Neurological progression speed (non-ambulatory over 7 days after symptom), n (%)	6(86)	7(23)	0.002
Tumor types (rapid growth), n (%)	1(14)	13(42)	0.17
The days from non-ambulatory to surgery (within 48 hours), n (%)	5(71)	15(48)	0.27

There were no significant differences in age, tumor type, visceral organ metastasis, neurological grade, or time to surgery at the non-ambulatory stage. Details of patients who recovered independent ambulation are summarized in Table 3.

**Table 3:** Detail of patients with recovered independent ambulation. F, female; M, male; y, year.

	Tumor type	Modified Frankel grade	Past history of therapy	Visceral organ metastasis	The days from symptom to non-ambulation	The days from non-ambulation to surgery	Survival days after surgery
74y F	lung	B	-	-	10	1	567
69y M	kidney	C1	+	-	42	2	1026
54y F	lung	C1	-	+	6	2	482
78y M	prostate	C1	-	-	14	2	unknown
78y M	lung	C2	-	-	12	2	251
78y F	colon	C2	-	+	11	9	unknown
68y F	leiomyosarcoma	C2	-	-	9	0	1335

## Discussion

While several studies have reported surgical outcomes in non-ambulatory patients with spinal metastasis, the recovery rate of daily life activities with independent ambulation remains uncertain. Our study showed that while the recovery rate was low, patients with no history of systematic therapy or slow progression of paralysis tended to recover independent ambulation postoperatively, even if they were in a non-ambulatory condition due to metastatic spinal cord compression.

A randomized controlled trial by Patchell et al. reported that the recovery rate of ambulation in patients unable to walk at the time of study enrollment was 62% (10/16) after surgery [2]. They defined ambulation as walking for four steps, even if a cane or walker was required. Other studies also reported several ambulation recovery rates using different outcome measures at different time points. They defined ambulation by several criteria (Frankel D, ASIA D, 10-m walking, walking for a few steps, or not mentioned), and this variety of ambulation definitions created ambiguity in the interpretation of the findings and their application to patient care [5]. We could not know whether the improvement in walking ability translated to an improvement in quality of life. To better understand the surgical results, we used two outcomes: the independent ambulation rate and the neurological recovery rate. When we defined ambulation as independent walking ability, the recovery rate of 18% was lower than those in previous reports of recovered ambulation rates (30-62%) [2,4]. However, neurological improvement was confirmed in 58% of the patients, and the outcome was within the range of previous findings. These data indicated that, in the real world, most patients could not spend their daily lives independently once they reached the non-ambulation stage, despite having neurological improvement after surgery. Therefore, determining the treatment plan before progression to the non-ambulated stage is important for patients with metastatic spinal metastases. Several trials have been reported for early diagnosis and treatment, including a rapid referral system to MRI scanning and oncology specialists and the encouragement of self-referral to the hospital when severe back pain occurs [9-11]. These trials reported the effect of reducing the proportion of patients who were unable to walk on the day of the MRI scan. Establishing such a system to detect at-risk patients earlier would be important to reduce the number of patients who are unable to walk due to metastatic spinal cord compression.

Several prognostic factors have been reported for neurological recovery. Among them, neurological grade and the time of surgery are important parameters [12-15]. If there is neurological deterioration due to spinal cord compression, early surgery is recommended before the neurological deficit progresses [14]. When we defined the outcome as independent gait, there were no significant differences in neurological grade or the time of surgery, which might be explained by the small number of patients, the selection of those with paralysis progression (only non-ambulatory), and the fact that most surgeries were performed early (within 48 hours after the patients were unable to walk). The speed of paralysis progression and the presence or absence of a history of systematic therapy were significant prognostic factors in our study, indicating that disease progression speed and the expected impact of systematic therapy are important predictors of postoperative ambulation stage. If paralysis progression is slow and the effect of systematic therapy is expected, patients will return to ambulatory daily life. On the other hand, we could not expect much of the paralysis to progress rapidly despite the presence of systematic therapy. The purpose of surgery is to gain independent gait, relieve pain, gain spinal stability, and prevent paralysis. There would be an indication for surgery, even in patients who were not expected to be able to walk. However, considering the short life expectancy and the high rate of complications, the indication for surgery would be uncertain for patients with poor outcomes. We could not report the survival within half a year after surgery for patients with recovered ambulation in this study (Table 3). Considering the poor condition due to disease progression and the time required to recover from a neurological deficit, half a year of survival was too short to return to daily life with independent ambulation. Survival prediction would also be a determinant of surgical indications.

The limitations of this study were its small sample size and patient selection criteria. Our study included only patients with spinal cord compression at cervical and thoracic sites. We did not include patients who could not walk because of radiculopathy, back pain, or lumbar lesions. Therefore, our findings were applicable only to cases of severe paralysis with metastatic spinal cord compression. Second, regarding prognostic factors, we could not investigate specific tumor factors because of the variety of tumor types. Chemosensitivity and tumor growth speed are important factors that differ by tumor type. A larger case series is needed to specifically investigate the prognostic factors associated with tumor type. Finally, the period of our study was approximately 15 years (2004-2020), and most patients did not undergo recently developed targeted molecular therapies. Recently, several second- and third-line treatments have been developed, and patients’ sensitivity to systematic therapy has improved. While our results showed poor outcomes for patients with past systematic therapy, surgical outcomes may improve with the development of targeted molecular therapies.

## Conclusions

The recovery to independent ambulation after surgery for non-ambulatory patients with metastatic spinal cord compression was poor, despite having neurological improvement. Therefore, determining the treatment plan before progression to the non-ambulated stage is important for patients with metastatic spinal metastases. Even though the paralysis progresses to the non-ambulatory stage, the recovered independent ambulation could be expected in patients with slow progression of paralysis or no history of systematic therapy.
